# Survival After Development of Contralateral Breast Cancer in Korean Patients With Breast Cancer

**DOI:** 10.1001/jamanetworkopen.2023.33557

**Published:** 2023-09-14

**Authors:** Hakyoung Kim, Tae In Yoon, Seonok Kim, Sae Byul Lee, Jisun Kim, Il Yong Chung, Beom Seok Ko, Jong Won Lee, Byung Ho Son, Sungchan Gwark, Jeong Kyeung Kim, Hee Jeong Kim

**Affiliations:** 1 Department of Surgery, Dongguk University College of Medicine, Dongguk University Ilsan Hospital, Goyang, Republic of Korea; 2 Division of Breast Surgery, Department of Surgery, Dongnam Institute of Radiological and Medical Science, Busan, Republic of Korea; 3 Department of Clinical Epidemiology and Biostatistics, College of Medicine, University of Ulsan, Asan Medical Center, Seoul, Republic of Korea; 4 Division of Breast Surgery, Department of Surgery, University of Ulsan College of Medicine, Asan Medical Center, Seoul, Republic of Korea; 5 Department of Surgery, Ewha Woman’s University College of Medicine, Ewha Woman’s University Mokdong Hospital, Seoul, Republic of Korea; 6 Maria Breast Clinic, Daegu, Republic of Korea

## Abstract

**Importance:**

Contralateral breast cancer (CBC) is the most frequently diagnosed primary cancer in patients with breast cancer. Although many studies have reported survival after the development of CBC, results have been inconsistent.

**Objective:**

To investigate whether the development of CBC is associated with survival among patients with breast cancer.

**Design, Setting, and Participants:**

This cohort study was conducted at the Asan Medical Center, Korea, among patients who were diagnosed with primary unilateral, nonmetastatic, stage 0 to III breast cancer between 1999 and 2013 and followed up through 2018. The median (IQR) follow-up was 107 (75-143) months. Patients were categorized into CBC and no-CBC groups by whether they developed CBC during the follow-up period. Data were analyzed from November 2021 to March 2023.

**Exposure:**

Development of CBC.

**Main outcomes and measures:**

Survival rates of CBC and no-CBC groups were compared using a time-dependent Cox proportional hazard model in the entire study population and in subgroup analyses by interval of CBC development and subtype of the primary breast cancer.

**Results:**

Among 16 251 patients with breast cancer (all Asian, specifically Korean; mean [SD] age, 48.61 [10.06] years), 418 patients developed CBC. There was no significant difference in overall survival between CBC and no-CBC groups (hazard ratio, 1.166; 95% CI, 0.820-1.657). Patients who developed CBC within 1.5 years after the surgery of the primary breast cancer had a higher risk for overall death during the study period (hazard ratio, 2.014; 95% CI, 1.044-3.886), and those who developed CBC after 1.5 years showed no significant difference in survival compared with the no-CBC group. Patients with hormone receptor (HR)–positive and human epidermal growth factor receptor 2 (*ERBB2*, formerly HER2)–negative breast cancer had a higher risk for overall death in the CBC group (hazard ratio, 1.882; 95% CI, 1.143-3.098) compared with the no-CBC group.

**Conclusions and Relevance:**

This study found that development of CBC in patients with breast cancer was not associated with survival but that early development of CBC after diagnosis of the primary breast cancer or development of CBC in patients with HR-positive/*ERBB2*-negative breast cancer was associated with survival. These results may provide valuable information for patients seeking advice on opting for contralateral prophylactic mastectomy.

## Key Points

**Question:**

Is the development of contralateral breast cancer associated with survival among patients with breast cancer?

**Findings:**

In this cohort study among 16 251 patients with stage 0 to III breast cancer, those who developed contralateral breast cancer showed no significant difference in overall survival compared with patients who did not develop this cancer.

**Meaning:**

These results may provide valuable information for counseling patients who are considering an option for prophylactic contralateral mastectomy.

## Teaser

This cohort study investigates the association of contralateral breast cancer with survival among Korean patients with breast cancer.

## Introduction

Contralateral breast cancer (CBC) is the most common primary cancer among patients with breast cancer.^1,2^ The cumulative incidence of CBC constantly increases after the primary breast cancer (PBC) diagnosis with longer follow-up; the reported annual incidence of CBC ranges from 0.2% to 0.7%.^1,3,4,5^ Our 2019 study^6^ with 8719 patients diagnosed with breast cancer 1989 to 2008 from the Asan Medical Center, Korea, presented consistent results, with a 10-year cumulative incidence of 6.1% in patients aged less than 35 years and 2.3% in those aged 35 years or older.

Many previous studies have analyzed whether the development of CBC in patients with breast cancer was associated with survival. However, results were inconsistent; some studies showed inferior survival in patients with CBC,^1,3,7^ while other studies showed no significant difference.^8,9,10^ Langballe et al^1^ reported in a nationwide population study in Denmark that patients with breast cancer who developed CBC had a more than 2-fold higher rate of breast cancer–specific death compared with patients who did not develop CBC and that a short interval between PBC and CBC was an important factor independently associated with worse survival. Verkooijen et al^8^ reported in a population study in Geneva, Switzerland, that patients with unilateral breast cancer did not show significant survival differences compared with patients with metachronous or synchronous bilateral breast cancer.

The prognosis of patients with breast cancer after developing CBC is an important issue, especially for young patients with breast cancer, who have a higher chance of developing CBC. The reason for this is that they have longer life expectancy,^1,5^ and diagnosis of breast cancer at a young age is a risk factor associated with developing CBC.^11^ Meanwhile, the age distribution of Korean patients with breast cancer is younger compared with populations in Western countries.^12,13^ However, the few Korean or Asian studies dealing with the association of CBC with survival show varying outcomes.

Therefore, the aim of this study was to investigate whether the development of CBC in Korean patients with breast cancer was associated with survival. We compared overall survival (OS) and breast cancer–specific survival (BCSS) between patients with breast cancer segregated by the development of CBC. We also performed a subgroup analysis to investigate whether survival differed in those at a higher risk of developing CBC.

## Methods

This cohort study was approved by the Asan Medical Center Review Board, which waived informed consent because the study was based on retrospective clinical data. This study followed the Strengthening the Reporting of Observational Studies in Epidemiology (STROBE) reporting guideline for observational studies.

### Study Population

This retrospective, single-center study used the Asan database, a prospectively collected archive of patients with breast cancer treated at the Asan Medical Center, Korea. The database provided patient information, pathology reports, types and modality of treatment, and recurrence and survival status.

We included patients diagnosed with stage 0 to III breast cancer between January 1999 and December 2013. Follow-up for the development of CBC, recurrence, and death was done until December 2018, and the median (IQR) follow-up duration was 107 (75-143) months. We excluded patients with other malignant breast diseases, such as malignant phyllodes tumor or lymphoma, stage IV breast cancer, synchronous bilateral breast cancer, and male breast cancer. Synchronous bilateral breast cancer was defined as CBC detected simultaneously with PBC or within 6 months after the diagnosis of PBC. Patients with an unknown subtype of breast cancer, those who did not undergo surgery, and those without follow-up were also excluded (eFigure in Supplement 1).

### Assessment

Patients who met the criteria were divided into CBC and no-CBC groups based on the development of CBC during follow-up. The diagnosis of CBC was defined as the first occurrence of cancer more than 6 months after surgery for PBC. Patients who developed CBC after local, regional, or distant recurrence of the PBC were classified as the no-CBC group, and the follow-up was censored at the time of recurrence. We obtained clinicopathological information on the PBC, such as patient age at surgery, body mass index (BMI; calculated as weight in kilograms divided by height in meters squared), family history, breast cancer gene (*BRCA*) mutation status, pathology reports, treatment modality, recurrence status, cause of death, and follow-up period. Similar data on CBC were collected from the CBC group.

Our primary outcomes were OS and BCSS compared between CBC and no-CBC groups. Follow-up for survival was defined as the time from the PBC surgery to ensuing events, whichever occurred first: death, last hospital visit confirming patient survival before December 2018, or a maximum of 17 years of follow-up. Our secondary outcome was survival compared between CBC and no-CBC groups by subgrouping them based on the time interval between surgeries of PBC and CBC, age of PBC surgery, and subtype of PBC.

### Pathology

Pathological data were evaluated at the Department of Pathology at the Asan Medical Center. We used pathological tumor-node-metastasis (TNM) staging for patients who had upfront surgery and clinical TNM staging for those who underwent neoadjuvant chemotherapy. TNM stage was assigned according to the American Joint Committee on Cancer (AJCC) classification system in the *AJCC Cancer Staging Manual* 7th edition. Tumor subtypes were categorized according to hormone receptor (HR) and human epidermal growth factor 2 (*ERBB2*, formerly HER2) status as HR-positive/*ERBB2*-negative (HR+/*ERBB2*−), HR-positive/*ERBB2*-positive (HR+/*ERBB2*+), HR-negative/*ERBB2*-positive (HR−/*ERBB2*+), and HR-negative/*ERBB2*-negative (HR−/*ERBB2*−) cancer. HR was considered positive if estrogen or progesterone receptors were positive. Immunohistochemistry was used to determine estrogen and progesterone HR and *ERBB2* status. Estrogen and progesterone receptor statuses were considered positive if more than 10% of cells were positive. For* ERBB2* overexpression, patients with immunohistochemistry grades 0 and 1+ were considered negative, and those with grade 3+ were considered positive. Grade 2+ cases were further evaluated using fluorescence in situ hybridization.

### Statistical Analysis

When comparing clinicopathological variables between CBC and no-CBC groups, the χ^2^ test was used for categorical variables and the Mann-Whitney U test for continuous variables. To estimate the survival rate of CBC as a time-dependent covariate, we used the Simon and Makuch method. A Cox regression model with time-dependent covariates was used to assess the association between CBC and survival outcomes. Variables with *P* < .2 in univariate analysis were included as adjusted covariates (age at surgery, BMI, year and type of surgery, histologic grade, subtype, T and N stages, adjuvant chemotherapy, and hormone therapy statuses). All covariates were based on the PBC. The association of CBC with survival in subgroups was evaluated by the interaction of CBC with the subgroup, which was visualized with a forest plot. A 2-sided *P* < .05 was considered statistically significant. All statistical analyses were performed using SAS statistical software version 9.4 (SAS Institute) and R statistical software version 3.6.1 (R Project for Statistical Computing). Data were analyzed from November 2021 through March 2023.

## Results

### Patient Characteristics

We collected data from 18 435 patients, and after exclusion, 16 251 patients (all Asian, specifically Korean; mean [SD] age, 48.61 [10.06] years) were included in the analysis. The flow for study population is shown in the eFigure in Supplement 1. In the study population, 418 patients (2.57%) developed CBC during the follow-up period and 15 833 patients (97.43%) did not. The median (IQR) follow-up duration was 107 (75-143) months. We compared clinicopathological characteristics of PBC between CBC and no-CBC groups (Table 1). Patients in the CBC group were younger than those in the no-CBC group (mean [SD] age, 44.68 [9.71] years vs 48.72 [10.05] years; *P* < .001). The CBC group compared with the no-CBC group had a higher percentage of patients with a first- or second-degree family history of breast cancer (68 patients [16.43%] vs 1521 patients [9.74%]; *P* < .001), nuclear grade 3 breast cancer (157 patients [43.13%] vs 5280 patients [36.59%]; *P* = .001), histologic grade 3 breast cancer (171 patients [50.00%] vs 5128 patients [37.23%]; *P* < .001), HR−/*ERBB2*− breast cancer (116 patients [27.75%] vs 2706 patients [17.09%]; *P* < .001), and T0 stage of disease (58 patients [13.88%] vs 1401 patients [8.85%]; *P* = .001). Approximately 10% of all patients (1506 patients [9.27%]) underwent the *BRCA* test; among them, a higher proportion of patients in the CBC group had *BRCA* mutations than in the no-CBC group (36 patients [38.3%] vs 183 patients [12.96%]; *P* < .001).

**Table 1.** Baseline Characteristics of Patients Based on First Breast Cancer

Characteristic	Patients, No. (%)			*P* value
	Total (N = 16 251)	No CBC (n = 15 833)	CBC (n = 418)	
Age, y				
Mean (SD), y	48.61 (10.61)	48.72 (10.05)	44.68 (9.71)	<.001
≤35	1318 (8.11)	1247 (7.88)	71 (16.99)	<.001
>35	14 933 (91.89)	14 586 (92.12)	347 (83.01)	
BMI				
<18.5	580 (3.69)	557 (3.64)	23 (5.56)	.08
18.5-24.9	10 863 (69.05)	10 574 (69.03)	289 (69.81)	
≥25	4289 (27.26)	4187 (27.33)	102 (24.64)	
Year of surgery (calendar period)				
1999-2004	3070 (18.89)	2941 (18.58)	129 (30.86)	<.001
2005-2009	5831 (35.88)	5654 (35.71)	177 (42.34)	
2010-2013	7350 (45.23)	7238 (45.71)	112 (26.79)	
Family history^a^				
No	14 444 (90.09)	14 098 (90.26)	346 (83.57)	<.001
Yes	1589 (9.91)	1521 (9.74)	68 (16.43)	
Histologic grade				
1	919 (6.51)	892 (6.48)	27 (7.89)	<.001
2	7896 (55.94)	7752 (56.29)	144 (42.11)	
3	5299 (37.54)	5128 (37.23)	171 (50)	
Nuclear grade				
1	1002 (6.77)	967 (6.7)	35 (9.62)	.001
2	8356 (56.48)	8184 (56.71)	172 (47.25)	
3	5437 (36.75)	5280 (36.59)	157 (43.13)	
Subtype				
HR+/*ERBB2*−	8951 (55.08)	8752 (55.28)	199 (47.61)	<.001
HR+/*ERBB2*+	2071 (12.74)	2026 (12.8)	45 (10.77)	
HR−/*ERBB2*+	2407 (14.81)	2349 (14.84)	58 (13.88)	
HR−/*ERBB2*−	2822 (17.37)	2706 (17.09)	116 (27.75)	
Stage T				
0 (in situ)	1459 (8.98)	1401 (8.85)	58 (13.88)	.001
1-2	13 892 (85.48)	13 559 (85.64)	333 (79.67)	
3-4	900 (5.54)	873 (5.51)	27 (6.46)	
Stage N				
0	10 656 (65.57)	10 361 (65.44)	295 (70.57)	.03
≥1	5595 (34.43)	5472 (34.56)	123 (29.43)	
Breast operation				
TM	6824 (41.99)	6613 (41.77)	211 (50.48)	<.001
BCS	9427 (58.01)	9220 (58.23)	207 (49.52)	
Radiotherapy				
No	5350 (33.04)	5183 (32.86)	167 (40.14)	.002
Yes	10 841 (66.96)	10 592 (67.14)	249 (59.86)	
Chemotherapy				
No	6441 (39.84)	6276 (39.84)	165 (39.76)	.97
Yes	9727 (60.16)	9477 (60.16)	250 (60.24)	
Hormone therapy				
No	5135 (31.82)	4947 (31.46)	188 (45.41)	<.001
Yes	11 002 (68.18)	10 776 (68.54)	226 (54.59)	
*BRCA* mutation (n = 1506)^b^				
No	1287 (85.46)	1229 (87.04)	58 (61.7)	<.001
Yes	219 (14.54)	183 (12.96)	36 (38.3)	

Abbreviations: BCS, breast-conserving surgery; BMI, body mass index (calculated as weight in kilograms divided by height in meters squared); *BRCA*, breast cancer susceptibility genes 1 and 2; CBC, contralateral breast cancer; *ERBB2*, human epidermal growth factor receptor 2 (formerly HER2); HR, hormone receptor; TM, total mastectomy.

^a^ First- and second-degree relative breast cancer family history.

^b^ 1506 of 16 251 patients underwent the *BRCA* test, and results are presented as percentages.

Among patients who developed CBC, we compared tumor characteristics of PBC and CBC (eTable in Supplement 1); 13 patients did not have pathological information for CBC, so data from 405 patients were analyzed. The median (IQR) interval for CBC development was 64 (34-101) months. The composition of subtypes was significantly different between PBC and CBC; the percentage of the HR+/*ERBB2*− subtype decreased from 110 patients (41.67%) in PBC to 82 patients (31.06%) in CBC (*P* = 0.01), whereas *ERBB2*+ subtypes regardless of HR status increased from 68 patients (25.76%) to 93 patients (35.23%) (*P* = 0.01). CBC tended to have lower T and N stages than PBC; the proportion of T0 stage increased from 58 patients (14.46%) to 105 patients (26.18%) (*P* < .001), and the proportion of N0 stage increased from 276 patients (70.95%) to 336 patients (86.38%) (*P* <.001) in CBC.

### Survival Analysis Comparing CBC and No-CBC Groups

**Figure 1.**
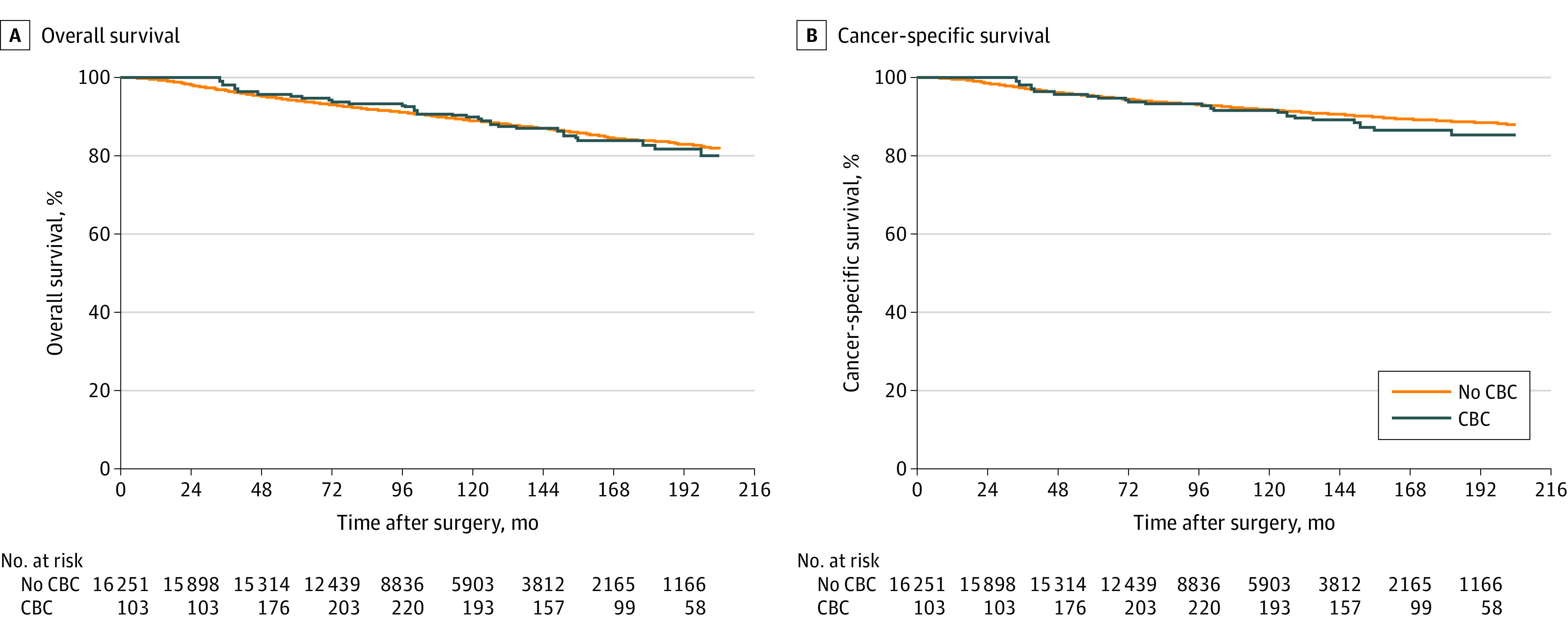
Survival Analysis Survival comparing contralateral breast cancer (CBC) and no-CBC groups is presented.

With a median follow-up of 107 months, 1761 patients died from any cause and 1282 patients died from breast cancer. We compared OS (hazard ratio, 1.166; 95% CI, 0.820-1.657; *P* = .39) and BCSS (hazard ratio, 1.357; 95% CI, 0.904-2.037; *P* = .14) using CBC as a time-dependent covariate and found no significant differences between CBC and no-CBC groups (Figure 1). In the multivariate analysis for OS and BCSS, there was no significant difference in survival between groups (Table 2).

**Table 2.** Multivariable Survival Analysis Using Cox Model With CBC as Time-Dependent Covariate

Outcome	Events, No./total No.		Crude		Adjusted^a^	
	No CBC	CBC	Hazard ratio (95% CI)	*P* value	Hazard ratio (95% CI)	*P* value
OS	1729/16 251	32/418	1.166 (0.820-1.657)	.39	1.001 (0.703-1.425)	>.99
BCSS	1258/16 251	24/418	1.357 (0.904-2.037)	.14	1.123 (0.747-1.690)	.58
*BRCA* subset (n = 1506)						
OS	191/1412	10/94	1.520 (0.797-2.901)	.20	1.290 (0.668-2.494)	.45
BCSS	149/1412	8/94	1.754 (0.851-3.614)	.13	1.495 (0.713-3.133)	.29

Abbreviations: BCSS, breast cancer–specific survival; *BRCA*, breast cancer susceptibility genes 1 and 2; CBC, contralateral breast cancer; OS, overall survival.

^a^ All were adjusted for age, body mass index (calculated as weight in kilograms divided by height in meters squared), year of surgery, type of surgery, histologic grade, subtype, T stage, N stage, adjuvant chemotherapy, and adjuvant hormone therapy. The *BRCA* subset was additionally adjusted for *BRCA*.

We performed separate multivariate analyses for OS and BCSS in the *BRCA* subset (patients who underwent the *BRCA* test), adjusting for *BRCA*. There were no significant differences in survival between groups regardless of *BRCA* status (OS: hazard ratio, 1.290; 95% CI, 0.668-2.494; *P* = .45; BCSS: hazard ratio, 1.495; 95% CI, 0.713-3.133; *P* = .29) (Table 2).

### Subgroup Analysis by Interval of CBC Development

**Figure 2.**
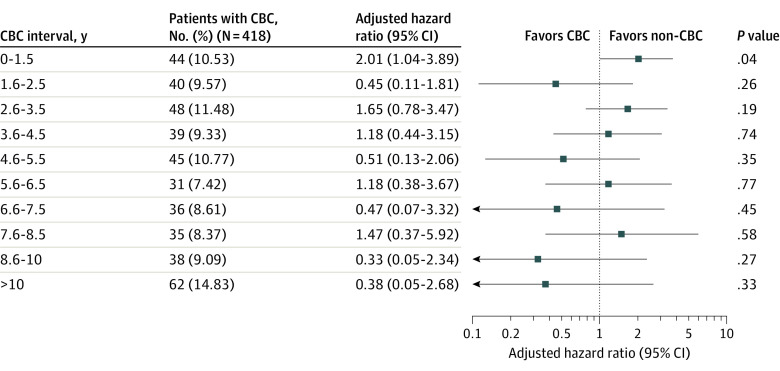
Overall Survival by Time Interval of Contralateral Breast Cancer (CBC) Development Analysis was adjusted for age, body mass index (calculated as weight in kilograms divided by height in meters squared), year of surgery, type of surgery, histologic grade, subtype, T stage, N stage, adjuvant chemotherapy, and adjuvant hormone therapy. All covariates were based on the primary breast cancer.

We divided patients in the CBC group according to the interval of CBC development and compared their OS with that of the no-CBC group. Given that patients who developed CBC within 6 months after the surgery of PBC were excluded, patients who developed CBC were divided by 1-year interval from 6 months since the surgery of PBC (for example, 0.5-1.5 years and 1.5-2.5 years). Patients who developed CBC within 1.5 years after the surgery for the PBC showed a 2.0 times higher risk of overall death compared with the no-CBC group after adjusting for covariates (hazard ratio, 2.014; 95% CI, 1.044-3.886; *P* = .04). Patients who developed CBC more than 1.5 years after the PBC surgery showed no significant difference in survival compared with patients in the no-CBC group (Figure 2).

### Subgroup Analysis by Age and Subtype of PBC

**Figure 3.**
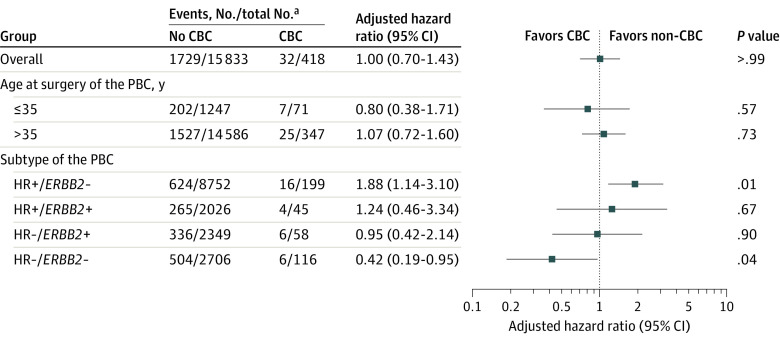
Overall Survival by Age and Subtype of Primary Breast Cancer (PBC) Analyses were adjusted for age, body mass index (calculated as weight in kilograms divided by height in meters squared), year of surgery, type of surgery, histologic grade, subtype, T stage, N stage, adjuvant chemotherapy, and adjuvant hormone therapy. All covariates were based on the PBC. CBC indicates contralateral breast cancer; *ERBB2*, human epidermal growth factor receptor 2; HR, hormone receptor. ^a^Event was overall death.

We subgrouped patients by age at PBC surgery (≤35 vs >35 years) and compared OS between CBC and no-CBC groups. There were 1318 patients (8.11%) aged 35 years or younger and 14 933 patients (91.89%) aged older than 35 years at the time of surgery for PBC. There was no significant difference in OS regardless of age group (≤235 years: hazard ratio, 0.803; 95% CI, 0.377-1.709; *P* = .57; >35 years: HR, 1.073; 95% CI, 0.721-1.597; *P* = .73) (Figure 3).

We categorized patients according to the subtype of PBC and compared OS between CBC and no-CBC groups. The HR+/*ERBB2*− subtype showed a 1.8-times higher risk of overall death in the CBC group (hazard ratio, 1.882; 95% CI, 1.143-3.098; *P* = .01). In the HR−/*ERBB2*− subtype, the CBC group had a 60% lower risk of overall death (hazard ratio, 0.425; 95% CI, 0.189-0.952; *P* = .04) (Figure 3).

## Discussion

In this large-scale, single-center, retrospective cohort study, we aimed to investigated the association of CBC development with survival in patients with breast cancer. From the Asan database, we analyzed 16 251 patients, and 2.57% of patients developed CBC, with a median follow-up of 107 months. We compared survival between CBC and no-CBC groups using the Cox regression model with time-dependent covariates and found no significant difference in OS or BCSS.

Comparing baseline characteristics of the PBC between CBC and no-CBC groups, the CBC group tended to have an earlier onset of breast cancer and a more favorable family history than the no-CBC group. In addition, the CBC group had a higher proportion of the HR−/*ERBB2*− subtype than the no-CBC group. Other studies had similar results,^11,14^ finding that diagnosis of PBC at a young age, a positive family history of breast cancer, and a triple-negative subtype were risk factors associated with developing CBC. These characteristics, however, overlap with baseline characteristics of patients with *BRCA*-mutation breast cancer.^15,16^ Furthermore, *BRCA* mutation was found to be a risk factor associated with developing CBC^11^; therefore, we assumed that there would be a larger proportion of patients with *BRCA* mutation in the CBC group than the no-CBC group even in patients who did not undergo the gene test. Given that we designed this study to investigate whether CBC development was associated with survival in the general population regardless of germline mutations, we wanted to adjust for *BRCA* mutation status in the analysis. Approximately 10% of our study population underwent the *BRCA* test; therefore, we performed separate multivariate analyses for OS and BCSS in this subset. However, no significant differences were observed in survival between CBC and no-CBC groups.

Patients who developed CBC in short intervals showed inferior OS in the CBC group compared with the no-CBC group. Similar results have been reported in previous studies. In a nationwide study using Surveillance, Epidemiology, and End Results (SEER) data,^17^ patients who developed CBC within 3 years of primary cancer diagnosis showed significantly worse OS compared with patients who did not develop CBC. A nationwide study conducted in the Netherlands reported similar results.^1^ Several factors have been proposed to explain the poor prognosis of early CBC. One explanation is that early presentation of CBC may be associated with metastasis soon after the diagnosis of CBC.^18^ Another explanation is that early CBC may have an unfavorable biological predisposition to worse survival.^17^ Third, there may have been missed bilateral breast cancers that became apparent 6 months after the PBC. Given that bilateral breast cancer is associated with worse survival, this could be 1 explanation for the poor prognosis.^19^ In our study, the number of patients who developed CBC within 1.5 years after the surgery was too small to analyze tumor characteristics, so in future studies recruitment of more patients will be needed. Lastly, early presentation of CBC may be associated with the development of resistance to treatment.^20^ Our study showed that a short interval for CBC development was a poor prognostic factor; however, there is no tool to identify patients at high risk for early CBC development, and future studies should focus on this.

A diagnosis of breast cancer at a young age is an important risk factor associated with CBC.^11^ Therefore, we aimed to investigate whether young patients were at a higher risk for mortality if they developed CBC. In most studies conducted in Western countries, the age set for defining young breast cancer is approximately 50 years, likely because the most frequent regional age group for breast cancer is ages 60 to 69 years.^21,22^ However, the age distribution of Korean patients with breast cancer differs considerably, with the most frequent age group for breast cancer being ages 40 to 49 years.^23^ Therefore, we categorized according to the age at PBC surgery by ages 35 years or younger vs older than 35 years, and approximately 8% of our study population was in the young breast cancer group. There was no significant difference in OS between the CBC and no-CBC groups regardless of age. Although 1 previous study^7^ found similar results, our study findings could be significant given that we applied an earlier age cutoff for subgrouping.

Subgroup analysis of the PBC subtype revealed inferior survival for the CBC group in the HR+/*ERBB2*− subtype. This may be associated with resistance to hormone therapy, which leads to worse survival. Further analysis of the treatment and matching of PBC with CBC tumor biology is warranted in future studies to investigate this association. In the HR−/*ERBB2*− subtype, patients with CBC showed superior survival. In our previous study^6^ comparing age-related risk factors associated with CBC, development of CBC in patients with the HR−/*ERBB2*− subtype peaked at approximately 10 years. Most recurrences or cancer-related deaths occur during the first 3 to 5 years in patients with the HR−/*ERBB2*− subtype.^24,25^ Given that patients who were diagnosed with recurrence before the development of CBC were censored at the time of recurrence, the CBC group consisted of patients who did not develop any recurrence in the early phase after PBC, thus substantiating the good prognosis. Despite these results, overall death numbers in each subgroup of the CBC group were too small, thus necessitating studies with larger populations.

### Limitations

Our study has several limitations, including a possible selection bias because the study was retrospective and single-centered. However, the Asan Medical Center is one of the largest hospitals in Korea and operates on approximately 10% of Korean patients with breast cancer; therefore, a large population study was possible. In addition, given that this was a single-center study, pathology and treatment were relatively uniform despite a long follow-up. Furthermore, although our study had a large study population, the number of patients or events, such as overall death, was too small to generalize our results, especially in subgroup studies. In the future, we could conduct a multicenter study with a larger population to avoid these limitations.

## Conclusions

In this cohort study, the occurrence of CBC was not associated with survival among Korean patients with breast cancer. However, the early development of CBC after the diagnosis of PBC or having a certain subtype of breast cancer were associated with survival. Such information may provide valuable counsel for patients considering contralateral prophylactic mastectomy.

## Notes

Supplement 1. **eFigure.** Study Flowchart **eTable.** Baseline Characteristics of Primary Breast Cancer and Contralateral Breast Cancer in Contralateral Breast Cancer Group

Supplement 2.
Data Sharing Statement
