# Missing Funding Information

**DOI:** 10.1001/jamanetworkopen.2023.48384

**Published:** 2023-12-11

In the Original Investigation titled “Survival After Development of Contralateral Breast Cancer in Korean Patients With Breast Cancer,”^1^ published September 14, 2023, the statement of study funding was missing from the Article Information section; the study was funded by grant HC20C0135 from the Korea Health Technology R&D Project through the Korea Health Industry Development Institute, funded by the Ministry of Health and Welfare, Republic of Korea.^1^ The article has been corrected.
