# Supplementary material for: Survival After Development of Contralateral Breast Cancer in Korean Patients With Breast Cancer
Source: JAMA Netw Open. 2023 Sep 14;6(9):e2333557. doi: 10.1001/jamanetworkopen.2023.33557 (PMC10502526; doi:10.1001/jamanetworkopen.2023.33557)
Supplement: Supplement 1. — eFigure. Study Flowchart eTable. Baseline Characteristics of Primary Breast Cancer and Contralateral Breast Cancer in Contralateral Breast Cancer Group [file jamanetwopen-e2333557-s001.pdf]

Supplemental Online Content

Kim H, Yoon TI, Kim S, et al. Survival after development of contralateral breast cancer in Korean patients with breast cancer. *JAMA Netw Open*. 2023;6(9):e2333557.
doi:10.1001/jamanetworkopen.2023.33557

eFigure. Study Flowchart
eTable. Baseline Characteristics of Primary Breast Cancer and Contralateral Breast Cancer in Contralateral Breast Cancer Group

This supplefmental material has been provided by the authors to give readers additional information about their work.

© 2023 Kim H et al. *JAMA Network Open.*

eFigure 1. Study Flow

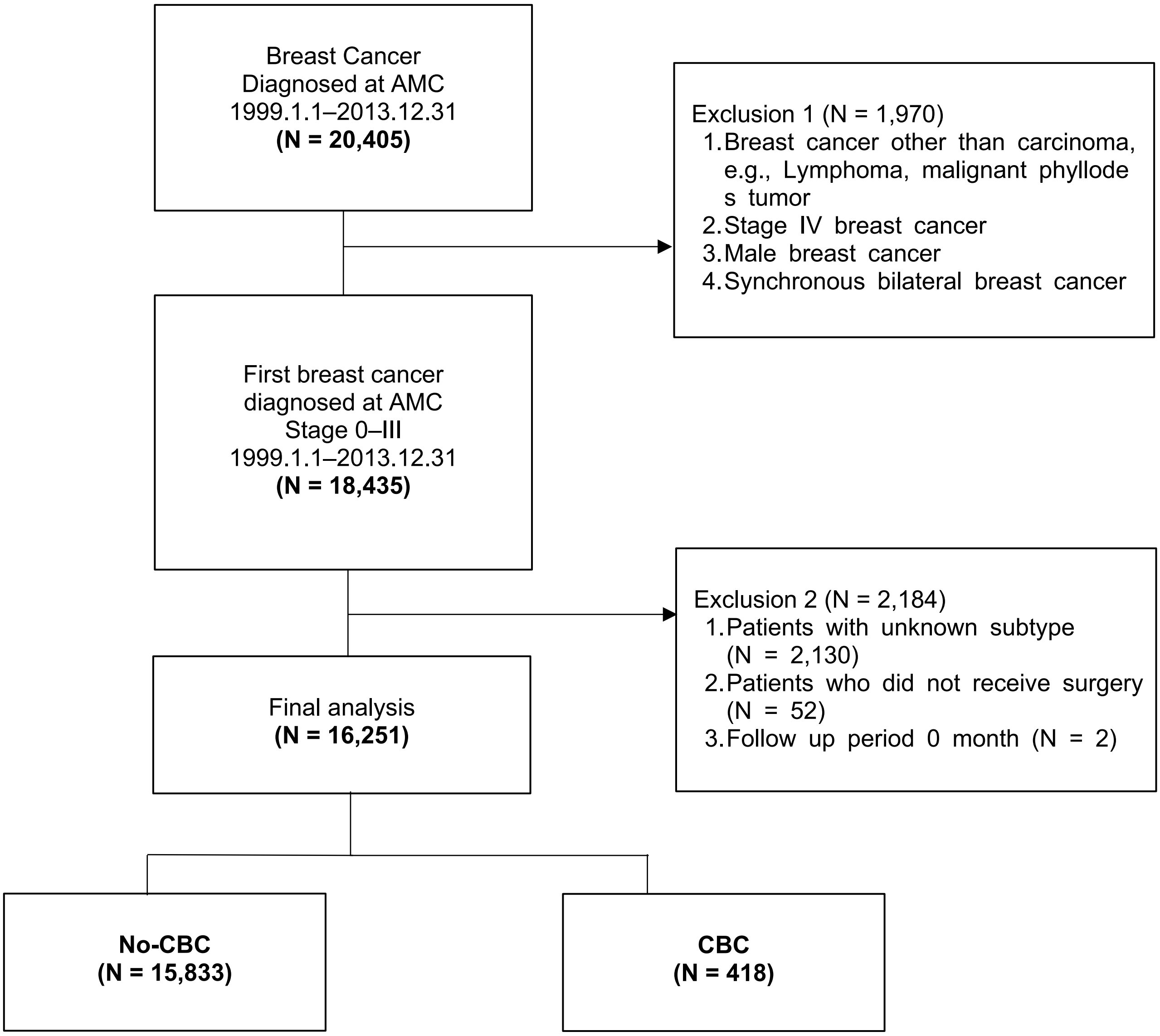

AMC: Asan Medical Center, CBC: contralateral breast cancer.

© 2023 Kim H et al. *JAMA Network Open.*

eTable 1. Baseline characteristics of the primary breast cancer and contralateral breast cancer in the contralateral breast cancer group.

	CBC patients (N=405)		p-value
	PBC	CBC	
	No. (%)	No. (%)	
Median CBC interval (months)	64 (IQR 34, 101)		
Mean Age ± SD	44.55±9.67	50.39±10.2	
Histologic grade			
1 or 2	152 (54.87)	155 (55.96)	0.761
3	125 (45.13)	122 (44.04)	
Nuclear grade			
1 or 2	226 (61.25)	227 (61.52)	0.927
3	143 (38.75)	142 (38.48)	
Subtype			
HR+/HER2-	110 (41.67)	82 (31.06)	0.011
HR+/HER2+	26 (9.85)	42 (15.91)	
HR-/HER2+	42 (15.91)	51 (19.32)	
HR-/HER2-	86 (32.58)	89 (33.71)	
Stage T			
0 (Tis)	58 (14.46)	105 (26.18)	<.001
1, 2	317 (79.05)	286 (71.32)	
3, 4	26 (6.48)	10 (2.49)	
Stage N			
0	276 (70.95)	336 (86.38)	<.001
≥1	113 (29.05)	53 (13.62)	
Chemotherapy			
No	159 (40.87)	245 (62.98)	<.001
Yes	230 (59.13)	144 (37.02)	
Hormone therapy			
No	177 (45.74)	167 (43.15)	0.391
Yes	210 (54.26)	220 (56.85)	

PBC, primary breast cancer; CBC, contralateral breast cancer; IQR, interquartile range; SD, standard deviation; HR, hormone receptor; HER2, human epidermal growth factor receptor 2.

© 2023 Kim H et al. *JAMA Network Open.*
