# Supplementary material for: Survival After Development of Contralateral Breast Cancer in Korean Patients With Breast Cancer
Source: JAMA Netw Open. 2023 Sep 14;6(9):e2333557. doi: 10.1001/jamanetworkopen.2023.33557 (PMC10502526; doi:10.1001/jamanetworkopen.2023.33557)
Supplement: Supplement 2. — Data Sharing Statement [file jamanetwopen-e2333557-s002.pdf]

Data Sharing Statement

Kim. Survival After Development of Contralateral Breast Cancer in Korean Patients With Breast Cancer. *JAMA Netw Open*. Published online September 14, 2023. doi:10.1001/jamanetworkopen.2023.33557

Data
Data available: No
